# Compromised nonsense-mediated RNA decay results in truncated RNA-binding protein production upon DUX4 expression

**DOI:** 10.1016/j.celrep.2023.112642

**Published:** 2023-06-13

**Authors:** Amy E. Campbell, Michael C. Dyle, Roberto Albanese, Tyler Matheny, Kavitha Sudheendran, Michael A. Cortázar, Thomas Forman, Rui Fu, Austin E. Gillen, Marvin H. Caruthers, Stephen N. Floor, Lorenzo Calviello, Sujatha Jagannathan

**Affiliations:** 1 Department of Biochemistry and Molecular Genetics, University of Colorado Anschutz Medical Campus, Aurora, CO 80045, USA; 2 Functional Genomics Research Centre, Human Technopole, 20157 Milan, Italy; 3 Computational Biology Research Centre, Human Technopole, 20157 Milan, Italy; 4 RNA Bioscience Initiative, University of Colorado Anschutz Medical Campus, Aurora, CO 80045, USA; 5 Department of Biochemistry, University of Colorado Boulder, Boulder, CO 80309, USA; 6 Department of Craniofacial Biology, University of Colorado Anschutz Medical Campus, Aurora, CO 80045, USA; 7 Medical Scientist Training Program, University of Colorado Anschutz Medical Campus, Aurora, CO 80045, USA; 8 Department of Cell and Tissue Biology, University of California, San Francisco, San Francisco, CA 94143, USA; 9 Helen Diller Family Comprehensive Cancer Center, University of California, San Francisco, San Francisco, CA 94143, USA; 10 These authors contributed equally; 11 Lead contact

## Abstract

Nonsense-mediated RNA decay (NMD) degrades transcripts carrying premature termination codons. NMD is thought to prevent the synthesis of toxic truncated proteins. However, whether loss of NMD results in widespread production of truncated proteins is unclear. A human genetic disease, facioscapulohumeral muscular dystrophy (FSHD), features acute inhibition of NMD upon expression of the disease-causing transcription factor, DUX4. Using a cell-based model of FSHD, we show production of truncated proteins from physiological NMD targets and find that RNA-binding proteins are enriched for aberrant truncations. The NMD isoform of one RNA-binding protein, SRSF3, is translated to produce a stable truncated protein, which is detected in FSHD patient-derived myotubes. Ectopic expression of truncated SRSF3 confers toxicity, and its downregulation is cytoprotective. Our results delineate the genome-scale impact of NMD loss. This widespread production of potentially deleterious truncated proteins has implications for FSHD biology as well as other genetic diseases where NMD is therapeutically modulated.

## In brief

Campbell et al. show that DUX4-induced loss of the RNA quality control pathway, nonsense-mediated RNA decay, results in widespread production of truncated proteins from physiological RNA targets. One such truncated protein, SRSF3-TR, contributes to DUX4-induced cytotoxicity.

## Graphical Abstract

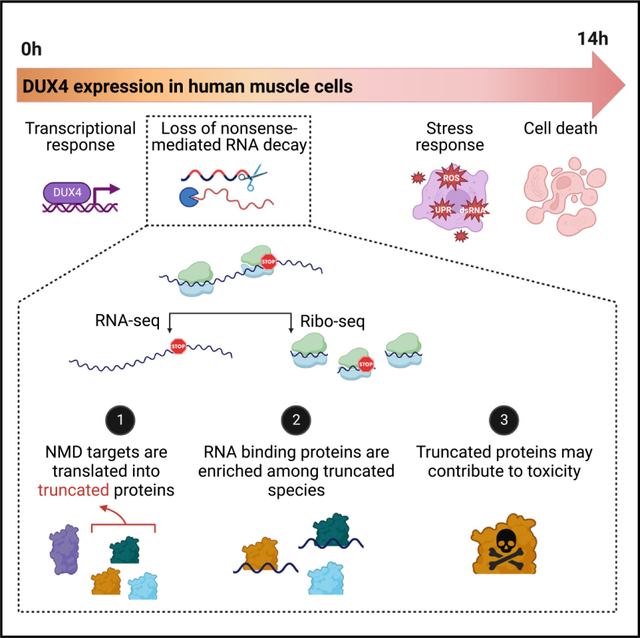

## INTRODUCTION

Nonsense-mediated RNA decay (NMD) degrades transcripts containing premature termination codons (PTCs) that arise from nonsense mutations or RNA processing errors. Through this mechanism, NMD prevents the production of potentially toxic truncated proteins.^1^ In addition to its role as a quality control mechanism, NMD also serves to regulate the expression of physiological transcripts that mimic NMD substrates.^1^ Such transcripts include a cassette exon containing a PTC, upstream open reading frames (ORFs), or long 3′ untranslated regions. Additionally, intricate auto- and cross-regulatory feedback loops utilize NMD to titrate the level of various splicing factors. An excess amount of these splicing factors facilitates the inclusion of a PTC-containing exon that reduces gene expression (“unproductive splicing”).^2,3^ Due to its dual role as a quality control and gene-regulatory mechanism, NMD efficiency is modulated in a variety of physiological contexts, including cell stress, differentiation, and development.^4–7^ NMD is also therapeutically targeted to allow production of certain truncated proteins that retain residual function to counter loss-of-function genetic diseases.^8^

Consistent with NMD’s role as a quality control mechanism, depletion of proteins involved in NMD,^9,10^ as well as pharmacological inhibition of NMD,^11,12^ can allow the production of truncated proteins from genes containing nonsense mutations. However, since NMD also destabilizes thousands of physiological aberrant transcripts,^1,13–15^ whether NMD loss generates truncated proteins on a broader level is not known. In mice, compromised NMD has been shown to be immunogenic,^16,17^ hinting at the production of truncated proteins with neoantigenic epitopes, although the identity of such proteins has not been characterized. It also remains an open question whether NMD inhibition has broad deleterious consequences for the cell.

In this study, we utilized a cellular model of a human genetic disease, facioscapulohumeral muscular dystrophy (FSHD), where NMD is naturally inhibited,^18^ to investigate the molecular and functional consequences of NMD loss. FSHD is a prevalent progressive myopathy caused by misexpression of a double homeodomain transcription factor, DUX4, in skeletal muscle.^19,20^ DUX4 is normally expressed during embryonic development, where it activates the first wave of zygotic gene expression.^21–23^ In individuals with FSHD, DUX4 is reactivated in muscle and induces apoptotic death leading to atrophy.^24–27^ We have shown that DUX4 misexpression in muscle cells causes rapid and acute NMD inhibition followed by proteotoxic stress and, eventually, translation inhibition.^18,28^

Here, we asked whether aberrant RNAs that accumulate upon DUX4-mediated loss of NMD produce truncated proteins by performing paired RNA sequencing (RNA-seq) and ribosome profiling (Ribo-seq) at 0, 4, 8, and 14 h following expression of DUX4 in MB135-iDUX4 human skeletal muscle myoblasts, a well-characterized cellular model of FSHD.^29,30^ While RNA-seq measures transcript abundance, Ribo-seq measures ribosome density along an mRNA.^31^ Thus, Ribo-seq serves as a proxy for active translation and allows delineation of translation start and end sites to characterize the protein products made from aberrant RNAs. Using Ribo-seq, we found that aberrant RNAs, which accumulate upon DUX4 expression, are actively translated to produce truncated proteins, particularly truncated RNA-binding proteins (RBPs) and splicing factors. We show that one such truncated splicing factor, serine/arginine-rich splicing factor 3 (SRSF3-TR), is expressed in FSHD muscle cell cultures and contributes to DUX4 toxicity. Thus, our findings indicate that loss of NMD results in widespread production of truncated proteins with deleterious cellular consequences.

## RESULTS

### DUX4 expression allows functional exploration of the consequences of compromised NMD

**Figure 1.**
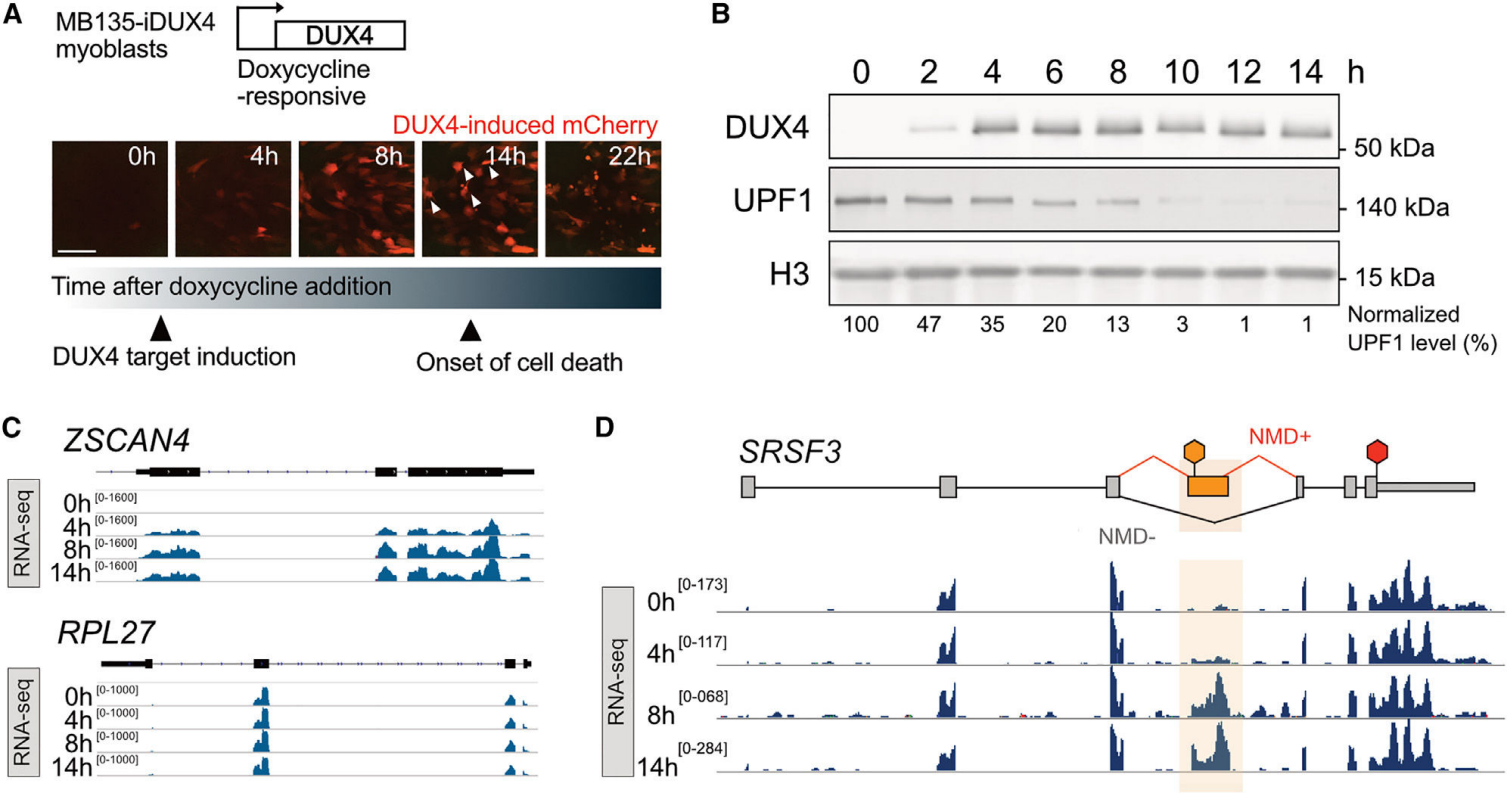
Synchronous expression of DUX4 in MB135-iDUX4 myoblasts enables time-course analyses of downstream gene expression changes (A) Time lapse images from live cell fluorescence microscopy of MB135-iDUX4/ZSCAN4-mCherry myoblasts following treatment with doxycycline to induce DUX4. Arrowheads indicate overtly dying cells. Scale bar, 150 μm. (B) Representative western blot analysis for DUX4, UPF1, and histone H3 (loading control) over a time course of DUX4 expression following doxycycline induction in MB135-iDUX4 myoblasts. (C) RNA-seq read coverage over a time course of DUX4 expression for DUX4 target gene *ZSCAN4* (top) and housekeeping gene *RPL27* (bottom). Data from replicate 1 of three replicates displayed. (D) RNA-seq coverage over SRSF3. The PTC-containing exon 4 is highlighted. The red hexagon indicates the normal stop codon, while the orange hexagon denotes the PTC. Data from replicate 1 of three replicates displayed. See also Video S1, Figure S1, and Table S1.

Misexpression of DUX4 in skeletal muscle cells inhibits NMD and induces cytotoxicity.^27,30,32^ To identify time points at which to measure transcript- and translation-level changes induced by DUX4 before the onset of overt cytotoxicity, we utilized a well-characterized doxycycline-inducible DUX4 human myoblast line, MB135-iDUX4,^30^ harboring a DUX4-responsive mCherry fluorescent reporter (Figure 1A). We live imaged these cells every 15 min for 28 h following doxycycline treatment to induce DUX4 (Figure 1A; Video S1). Expression of the DUX4-responsive mCherry was rapid and nearly synchronous, with fluorescence detection after 2 h. Cytotoxicity was first observed 9 h following DUX4 induction, with most cells dead or dying by 18 h (Video S1). Western blot analysis showed that levels of the key NMD factor UPF1 were reduced as early as 2 h post DUX4 induction and continued to decrease (Figure 1B). Two different markers of cell stress—*HSP5A* induction and eIF2α phosphorylation—show that loss of UPF1 protein and the drop in NMD efficiency occur prior to cell stress (Figures S1A–S1F), further confirming our previous observation that NMD inhibition is an early event during DUX4 expression.^18^ Given these data, we chose the time points of 4, 8, and 14 h post DUX4 induction to investigate the consequences of NMD loss.

First, we examined DUX4-induced transcriptome changes as measured by RNA-seq (Table S1A). As expected, transcripts of a DUX4 target gene, *ZSCAN4*, were absent in uninduced cells but highly expressed at 4 h and increased with time (Figure 1C, top), while the housekeeping gene *RPL27* was expressed throughout the time course (Figure 1C, bottom). Also as expected, aberrant transcript isoforms with PTC-containing exons, such as exon 4 of *SRSF3* (denoted as NMD+), were present at very low levels prior to DUX4 expression but increased in abundance thereafter, appearing as early as 4 h post induction (Figure 1D).

Genome-wide, DUX4 altered the expression of thousands of transcripts, with known DUX4 targets^29^ showing increasing upregulation throughout the time course (Figure S1G). Using K-means clustering, we grouped genes significantly altered (defined as absolute log2 fold change >1 and adjusted p value <0.01) at any point during the time course into five clusters (Figure S1H) and carried out Gene Ontology (GO) analysis on each cluster (Figure S1I; Tables S1B and S1C). The genes rapidly induced upon DUX4 expression (cluster 1) were enriched for negative regulation of cell differentiation, positive regulation of cell proliferation, and DNA-templated transcription, while those rapidly silenced upon DUX4 expression (cluster 5) were enriched for myogenesis, positive regulation of cell differentiation, and cytoskeleton organization. Together, this is illustrative of a general switch away from a differentiated muscle program and toward a proliferative phenotype, consistent with DUX4’s normal role in establishing an early embryonic program. Interestingly, the cluster of genes induced only at the late 14 h time point (cluster 3) was enriched for GO categories mRNA splicing, ribonucleoprotein transport, ubiquitin-dependent process, unfolded protein response, and hypoxia, which have all been previously reported as major signatures of DUX4-induced gene expression.^27,30,32^ Together, these RNA-seq data show that the 4, 8, and 14 h time points capture the temporal range of DUX4-induced gene expression changes and are consistent with early induction of transcriptional responses and late induction of cell stress response.

### Ribosome profiling shows concordance between transcript levels and ribosome occupancy upon DUX4 expression

Previous quantitative analysis of the DUX4-induced proteome via stable isotope labeling by amino acids in cell culture (SILAC) mass spectrometry showed discordant changes at the RNA versus protein level,^28^ raising the possibility that translation could be modulated upon DUX4 expression. Additionally, at later time points, DUX4 induces dsRNA-mediated activation of protein kinase R (PKR)^33^ and stimulates PKR-like ER kinase (PERK) via the unfolded protein response pathway,^28^ resulting in eIF2α phosphorylation, which is known to inhibit cap-dependent translation.^34^ Therefore, we asked whether transcript level changes driven by DUX4 expression were echoed at the level of translation by comparing the RNA-seq and Ribo-seq datasets.

**Figure 2.**
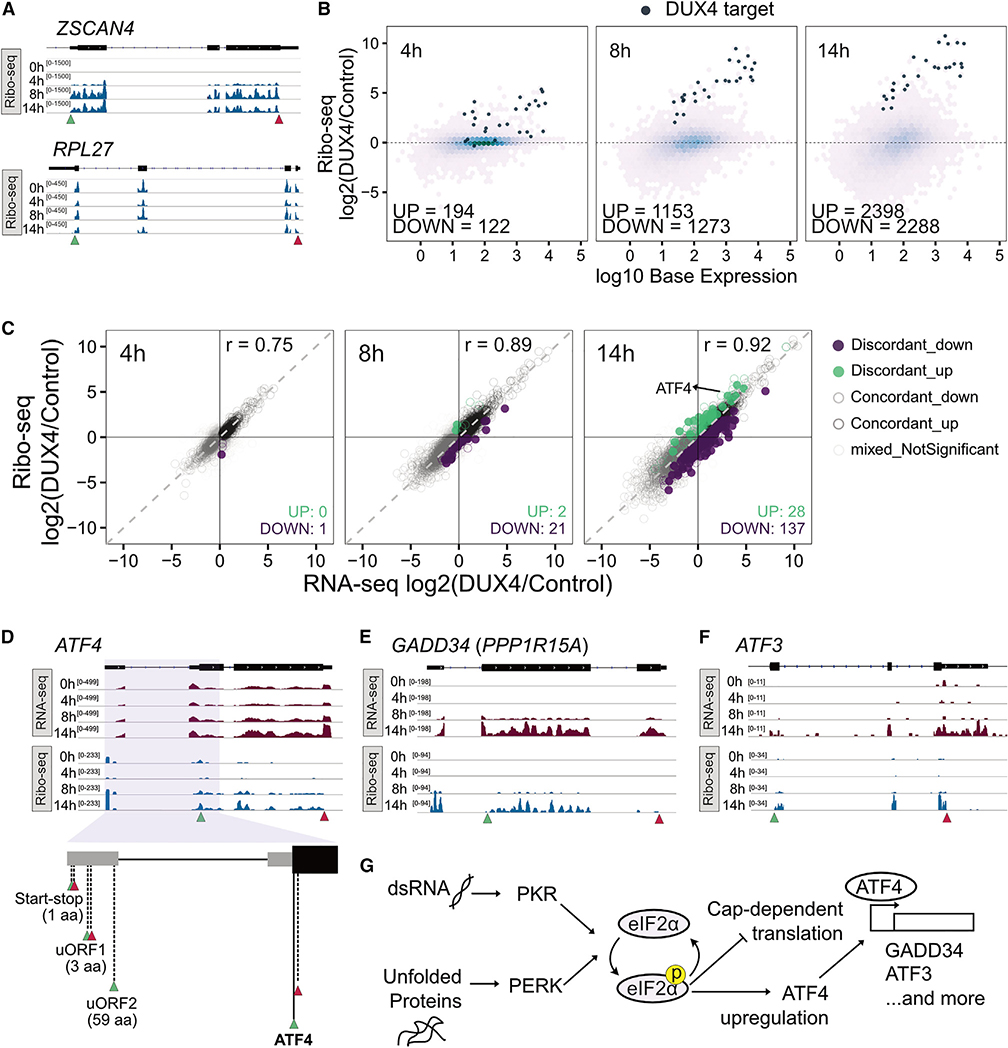
Ribo-seq shows high concordance between transcript levels and translation status (A) Ribo-seq read coverage over a time course of DUX4 expression for DUX4 target gene *ZSCAN4* (top) and housekeeping gene *RPL27* (bottom). Green triangle, translation start; red triangle, translation stop. Data from replicate 1 of three replicates displayed. (B) M-A plots for triplicate Ribo-seq data after 4, 8, and 14 h of DUX4 induction compared with the 0 h control. (C) Scatterplot of triplicate RNA-seq versus Ribo-seq log2 fold change after 4, 8, and 14 h of DUX4 expression. Significance defined as adjusted p value <0.01 for Ribo-seq fold change. (D) RNA-seq (top) and Ribo-seq (middle) coverage over *ATF4*; schematic showing the upstream ORF (uORF) and main ORFs of *ATF4* (bottom). (E and F) RNA-seq (top) and Ribo-seq (bottom) coverage over *GADD34* (E) and *ATF3* (F). Data from replicate 1 of three replicates displayed (D–F). (G) Schematic summary of how DUX4 expression influences translation and subsequent cell stress. See also Figure S2 and Table S2.

The characteristic three-nucleotide periodicity exhibited by the ribosome-protected RNA fragments confirmed the high quality of our Ribo-seq data (Figure S2). Representative Ribo-seq read coverage plots of the DUX4 target gene, *ZSCAN4*, showed no coverage in uninduced cells, low ribosome density beginning at 4 h, and active translation at 8 and 14 h (Figure 2A, top). In contrast, the housekeeping gene *RPL27* was translated throughout the time course (Figure 2A, bottom). The changes in ribosome association at these specific genes mirrored the differences seen in their mRNA levels (Figure 1C).

On a genome-scale, DUX4 altered the translation status for thousands of transcripts, with later time points showing larger differences and known DUX4 targets being translated at increasing levels throughout the time course (Figure 2B; Table S2A). Most genes were concordantly up- or downregulated at the level of transcript and inferred translation efficiency (Figure 2C; STAR Methods) at all time points with only a small number of genes showing some discordance. GO analysis of the discordantly regulated genes returned significant results only for the gene set that showed a mild translation downregulation at the 14 h time point (n = 137) with pathways such as protein targeting to endoplasmic reticulum (ER) and viral transcription being enriched, which, strikingly, were driven entirely by a group of ribosomal protein-encoding genes (Table S2B). This mild downregulation of translation is consistent with induction of the integrated stress response (ISR) pathway and DUX4-mediated eIF2α phosphorylation.^28,33^ A caveat of Ribo-seq to keep in mind is that it may not capture the true absolute change in translation efficiency induced by ISR, and therefore the translation downregulation we observe could be an underestimate.

Nonetheless, we observed several hallmarks of ISR activation at 14 h, including translation upregulation of *ATF4* (Figures 2C and 2D). Specifically, the start-stop regulatory element in the 5′ untranslated region of *ATF4*, which modifies downstream reinitiation,^35^ was highly occupied by ribosomes at 14 h (Figure 2D). The resultant upregulation of ATF4 protein can account for the subsequent transcriptional and translational induction of ATF4 targets *GADD34* and *ATF3* (Figures 2E and 2F). These data confirm the DUX4-induced phosphorylation of eIF2α via PKR and PERK activation culminate in a block in cap-dependent translation and stimulation of ISR signaling at the late 14 h time point (Figure 2G). However, in large part, DUX4-induced changes in transcript level are mirrored in their ribosome occupancy.

### DUX4 causes widespread truncated protein production

**Figure 3.**
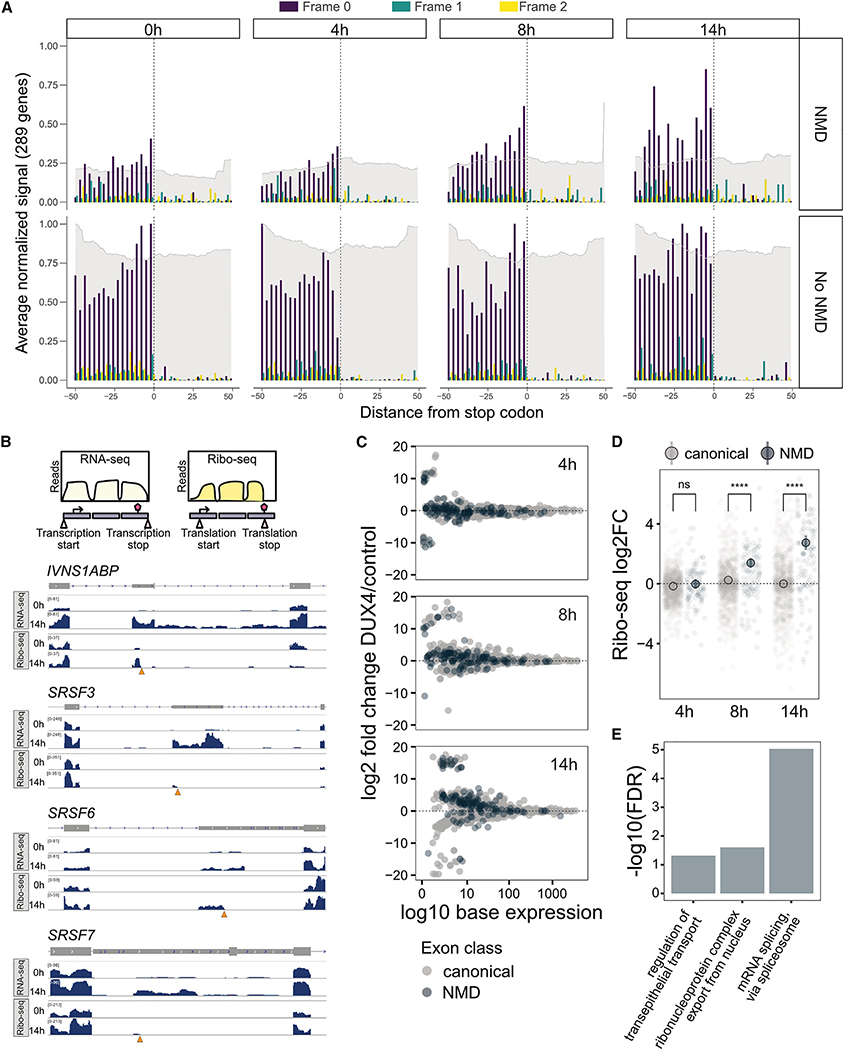
Exon-level analysis of denovo identified ORFs from Ribo-seq data shows translation of NMD-targeted aberrant RNAs (A) Normalized average of triplicate Ribo-seq profiles over stop codons pairs (PTCs and normal termination codons [NTCs]) from the same genes along DUX4 activation. Signal on different frames, as well as RNA-seq coverage, shown in different colors. (B) Schematic representation of paired RNA-seq and Ribo-seq experiment (top) and RNA-seq and Ribo-seq coverage over splicing-related genes *IVNS1ABP, SRSF3, SRSF6*, and SRSF7 (bottom). Orange triangles denote PTCs. Data from replicate 1 of three replicates displayed. (C) M-A plot of exon-level analysis of triplicate Ribo-seq data from 4, 8, and 14 h of DUX4 induction. The x axis represents mean expression calculated at the level of each exon within a gene. (D) Exon-level log2 fold change of triplicate Ribo-seq values at 4, 8, or 14 h of DUX4 expression for canonical and NMD exons. Statistical testing performed using two-sided Wilcoxon test. (E) GO analysis results of selected gene sets (biological process complete) for all NMD targets translated at 14 h. See also Figure S3 and Table S3.

Having shown that most transcripts induced by DUX4 are translated, and that NMD inhibition is an early consequence of DUX4 expression, we sought to determine whether truncated proteins are produced from PTC-containing RNAs that accumulate due to DUX4-mediated loss of NMD. To ask if and when aberrant RNAs are translated transcriptome-wide, we used ORFquant,^36^ a pipeline that identifies isoform-specific translation events from Ribo-seq data. We focused on detected ORFs ending at candidate PTCs in non-coding isoforms of protein-coding genes and monitored their translation levels across DUX4 induction. We observed ribosome footprint coverage at both canonical stop codons and PTCs with accurate frame resolution, with the expected drop in coverage after the stop codons (Figure 3A). Additionally, we detected an increase in ribosome footprints at PTCs, as well as a modest increase in RNA-seq signal (quantified in Figure S3E). When we compared the RNA-seq and Ribo-seq reads at candidate genes with NMD isoforms*, IVNS1ABP*, *SRSF3*, *SRSF6*, and *SRSF7*, we saw strong coverage across the PTC-containing exon with reads stopping at the PTC (Figures 3B and S3A–S3D). These results clearly indicate the translation of truncated proteins from NMD isoforms.

We then used DEXSeq^37^ to conduct exon-level differential analysis on the set of ORFquant-derived ORFs, using Ribo-seq data. This analysis identifies changes in relative exon usage to measure differences in the expression of individual exons that are not simply the consequence of changes in overall transcript level. After 4 h of DUX4 induction, 397 genes showed differential expression of specific exons, of which 24 are predicted NMD targets (Figure 3C), whereas later time points showed a greater number of exons (n = 96 at 14 h) that are unique to NMD targets as differentially expressed (Table S3A). We grouped exons based on their NMD target status and calculated their fold change in ribosome footprints at 4, 8, and 14 h of DUX4 expression compared with the 0-h time point (Figure 3D). We observed a progressive and significant increase in the translation status of NMD-targeted exons, but not canonical exons, at 8 and 14 h, confirming the translation of aberrant RNAs in DUX4-expressing cells.

To ask how the specific aberrant RNAs being translated in DUX4-expressing myoblasts might functionally affect cell homeostasis, we conducted GO analysis of the 74 truncated proteins being actively translated at 14 h of DUX4 induction (Figure 3E; Table S3B). Strikingly, the truncated proteins were enriched for genes encoding RBPs involved in mRNA metabolism and, specifically, splicing (Table S3B). Thus, not only do NMD targets accumulate upon DUX4 expression but they also lead to the translation of the truncated versions of many RBPs, which could have substantial downstream consequences to mRNA processing in DUX4-expressing cells.

### Truncated SRSF3 is present in FSHD myotubes and contributes to cytotoxicity

**Figure 4.**
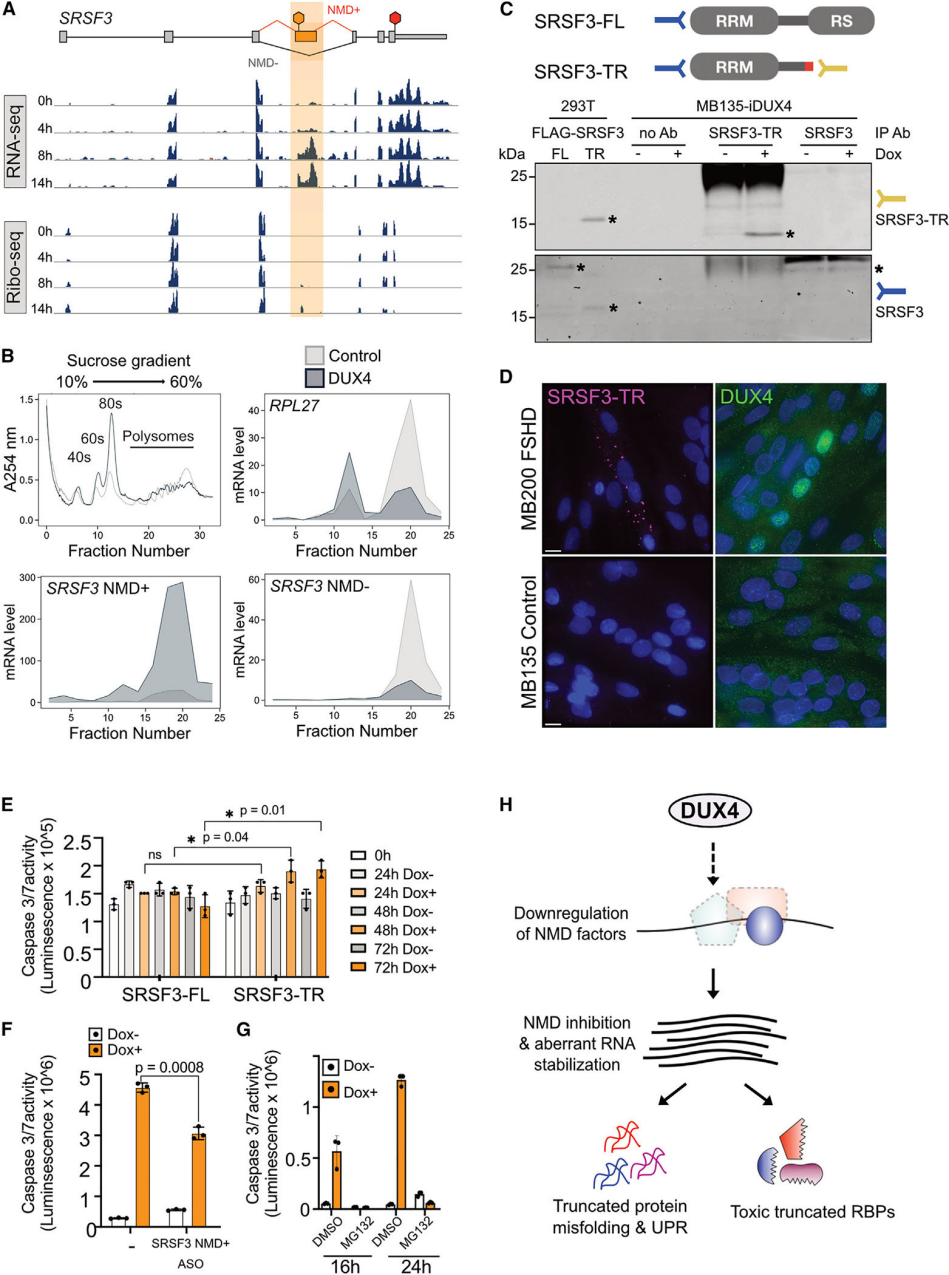
Truncated SRSF3 protein contributes to DUX4-induced cytotoxicity (A) RNA-seq and Ribo-seq coverage over *SRSF3*. PTC-containing exon 4 is highlighted. Red hexagon indicates the normal stop codon, while orange hexagon denotes the PTC. Data from replicate 1 of three replicates displayed. (B) Absorbance at 254 nm across a sucrose density gradient of lysates from control MB135-iDUX4 myoblasts and MB135-iDUX4 myoblasts expressing DUX4 for14 h (top left, n = 1). qRT-PCR measurement of *RPL27, SRSF3* NMD+, and *SRSF3* NMD− mRNA levels in DUX4-expressing myoblasts relative to control myoblasts from collected fractions (remaining panels, n = 3 technical replicates). (C) Detection of SRSF3 and SRSF3-TR in whole-cell extracts from 293T cells exogenously expressing FLAG-tagged full-length (FL) or truncated (TR) SRSF3 compared with protein lysates from MB135-iDUX4 myoblasts treated with (+) or without (−) doxycycline (Dox) to induce DUX4 and immunoprecipitated with a custom anti-SRSF3-TR antibody, no antibody (Ab), or a commercial SRSF3 antibody. IP, immunoprecipitation. Asterisks denote proteins of interest. Note the FLAG-tagged versions of SRSF3-FL and SRSF3-TR run at a higher molecular weight than the endogenous versions due to the epitope tag. Representative image of two biological replicates shown. (D) Representative immunofluorescence in MB135 control and MB200 FSHD myotubes differentiated for 72 h and stained with DAPI (blue) and anti-DUX4 (green) or custom anti-SRSF3-TR (pink) antibody. n = 1 biological replicate. Scale bar, 10 μm. (E) Caspase-3/7 activity in myoblasts expressing doxycycline (Dox)-inducible FL or TR SRSF3. (F) Caspase-3/7 activity following ASO-mediated knockdown of SRSF3 NMD+ in MB135-iDUX4 myoblasts left untreated (Dox−) or treated with doxycycline for 16 h (Dox+) to induce DUX4. (G) Cell viability measured by caspase-3/7 activity following co-treatment with doxycycline (Dox) to induce DUX4 and proteasome inhibition via MG132 for 16 or 24 h. (H) A working model where DUX4-induced downregulation of NMD factors leads to accumulation of aberrant RNAs producing truncated RBPs and misfolded proteins that trigger the unfolded protein response and toxicity. All error bars denote the standard deviation from the mean of three biological replicates, which are shown as individual data points. See also Figure S4.

To explore the role of truncated proteins in DUX4-induced cellular phenotypes, we chose SRSF3 for further characterization. SRSF3 is an serine/arginine (SR) family protein that possesses an N-terminal RNA-binding RNA recognition motif (RRM) and a C-terminal arginine/serine (RS)-rich domain responsible for protein-protein and protein-RNA interactions. The NMD isoform of SRSF3 encodes a truncated protein (SRSF3-TR) that lacks most of the RS domain and has been previously implicated in a variety of human pathologies.^38–42^ Examination of our RNA-seq and Ribo-seq data showed expression and translation of *SRSF3* NMD-targeted exon 4 that ends at the site of the PTC (Figure 4A).

To determine the translation status of the aberrant *SRSF3* transcript, we carried out polysome profiling using sucrose density gradient separation. The polysome profile after 14 h of DUX4 expression compared with control showed a higher fraction of 80S compared with polysomes (Figure 4B, top left). This is consistent with our prior observation of eIF2α phosphorylation^28,33^ and the resultant global downregulation of translation at 14 h. We extracted RNA from various fractions and profiled RNA levels of specific transcripts by qPCR. *RPL27* mRNA was predominantly ribosome bound in control cells, but this partially shifted to monosomes in DUX4-expressing cells (Figure 4B, top right). The NMD+ isoform of *SRSF3*, on the other hand, showed a massive increase in heavy polysomes in DUX4-expressing cells, consistent with its increased abundance at the RNA level (Figure 4B, bottom left). The *SRSF3* NMD− isoform showed a loss from the polysome fraction without a corresponding increase in the monosome fraction (Figure 4B, bottom right), suggesting that its regulation is primarily at the level of RNA abundance rather than at the level of translation. These data show that aberrant *SRSF3* mRNA is being actively translated into truncated protein in DUX4-expressing myoblasts and validate the ribosome footprints found on the NMD+ isoform.

To determine if we could stably detect truncated SRSF3 protein in cells, we generated an antibody recognizing a 10-amino-acid C-terminal neo-peptide unique to SRSF3-TR. This custom SRSF3-TR antibody was able to recognize FLAG-tagged SRSF3-TR exogenously expressed in 293T cells and endogenous SRSF3-TR immunoprecipitated from DUX4-expressing MB135-iDUX4 myoblasts (Figure 4C). We also used a commercial SRSF3 antibody that recognizes an N-terminal epitope common to both the full-length and truncated SRSF3. This antibody detected both exogenously expressed full-length and truncated FLAG-SRSF3, and a low level of endogenous full-length SRSF3, but was insufficient to visualize endogenous SRSF3-TR (Figure 4C), possibly due to lower affinity for this protein isoform in an immunoprecipitation assay. To complement this experiment, we carried out immunofluorescence for SRSF3-TR or DUX4 in differentiated FSHD and control muscle cells to determine if SRSF3-TR was present in FSHD myotubes expressing endogenous levels of DUX4. While there was no detectable SRSF3-TR staining in control cells, in DUX4-expressing FSHD cultures, SRSF3-TR appeared in cytoplasmic puncta (Figure 4D). Thus, not only is the *SRSF3* NMD+ isoform translated in DUX4-expressing cells, its protein product, SRSF3-TR can be detected in both DUX4-expressing MB135-iDUX4 myoblasts and in FSHD patient-derived myotubes.

To ask if expression of SRSF3-TR is deleterious to cells, we exogenously expressed FLAG-tagged full-length or truncated SRSF3 in healthy muscle cells. We found that SRSF3-TR, but not SRSF3-FL, reduced the viability of MB135 myoblasts (Figure 4E). To specifically knock down the SRSF3-TR isoform in DUX4-expressing myoblasts, we screened antisense oligonucleotides (ASOs) to identify one (a thiomorpholino 2′-deoxyribonucleotide 3′-thiophosphate oligonucleotide chimera^43,44^) that lowered the *SRSF3* NMD+ isoform without significantly affecting *DUX4* transcript level or target expression (Figure S4). Treatment of DUX4-expressing myoblasts with this thiomorpholino oligonucleotide resulted in a 34% reduction in cell death compared with untreated cells (Figure 4F). Finally, we found that blocking the proteasome, which is the primary mediator of NMD inhibition by DUX4,^18,28^ significantly rescued DUX4 toxicity (Figure 4G). These results suggest that truncated proteins confer toxicity to muscle cells via a gain-of-function mechanism. Significantly, this mechanism is potentially additive across the different species of truncated proteins that are produced upon DUX4 expression in myoblasts (Figure 4H).

## DISCUSSION

Loss of NMD leads to the stabilization of aberrant RNAs.^45^ However, it is not known whether these aberrant RNAs are translated and what proteins they might produce. Here, we paired RNA-seq and Ribo-seq across a time course of DUX4 expression in human skeletal muscle myoblasts to show that DUX4-induced loss of NMD causes truncated protein production at a genome level.

The production of truncated proteins upon perturbation of NMD by DUX4 has implications at both molecular and functional levels (Figure 4H). Protein truncation could result in a dominant-negative function that inhibits the activity of the remaining, cell-critical full-length protein. Truncated proteins might misfold and facilitate formation of protein aggregates, and some truncated proteins contain unique C-terminal extensions that could serve as neoantigens and might induce inflammation. It is important to note that these truncated proteins, while produced, could be highly unstable or rapidly cleared by the proteasome and not present at quantities significant enough to have a functional consequence. Nonetheless, when NMD is modulated as a therapeutic intervention for genetic diseases, it is important to consider whether truncated proteins are produced and whether this might have a negative impact on the cell. In physiological contexts where NMD efficiency is suppressed without deleterious consequences, cells may possess mechanisms that counter truncated protein production. Further investigation of the suppression or tolerance of truncated proteins could reveal mechanisms that enable the protein quality control rheostat to be adjusted to deal with variable NMD efficiencies.

In FSHD, there is evidence for truncated proteins contributing to myotoxicity via all the above mechanisms. Here, we show gain-of-function toxicity for SRSF3-TR. Prior work has demonstrated protein aggregation^33,46,47^ as well as immune cell infiltration^48–51^ in FSHD muscle. Our results suggest that these effects could be due to truncated proteins and neoantigenic epitopes. In addition, many of the identified DUX4-induced truncated proteins are RBPs and splicing factors. It is well established that DUX4 alters RNA splicing,^27,28,30,32^ and therefore it is interesting to speculate that truncated RBPs and splicing proteins might be responsible for inducing global RNA processing defects.^52^ Such misprocessing would generate aberrant RNAs that could act to further overwhelm the already inhibited NMD pathway.

In summary, we provide support for the widely held assumption that loss of NMD results in production of truncated proteins with deleterious cellular consequences. In doing so, we provide a framework to interpret the multifaceted phenotypes observed in FSHD as a potential result of NMD inhibition. Our findings provide a critical missing piece in the understanding of this essential quality control mechanism in both disease and physiology, which has implications for the treatment of genetic diseases.

### Limitations of the study

One limitation of our study with regard to drawing broad conclusions about the consequences of NMD inhibition is that DUX4-induced loss of NMD is potent, with multiple critical NMD factors undergoing proteolysis.^18,28^ Therefore, it is important to keep in mind the extent of loss of NMD in a particular system while extrapolating our results from DUX4-expressing cells. We also acknowledge that the number of truncated proteins we identify as translated based on exon-level analysis is likely an underestimate as many NMD targets contain a premature stop codon too close to the 5′ boundary of the NMD-inducing exon to reliably analyze Ribo-seq read coverage at the exon level. Another caveat is that our experiments show the global translation of truncated proteins but not that all truncated proteins are stably present at meaningful levels besides SRSF3-TR. Future experiments are needed to characterize the activity of each of the identified truncated proteins.

## STAR★METHODS

Detailed methods are provided in the online version of this paper and include the following:

### RESOURCE AVAILABILITY

#### Lead contact

Further information and requests for resources and reagents should be directed to and will be fulfilled by the lead contact, Sujatha Jagannathan (sujatha.jagannathan@cuanschutz.edu).

#### Materials availability

All unique reagents generated in this study are available from the lead contact with a completed Material Transfer Agreement. Plasmids generated in this study have been deposited to Addgene (plasmid #171951, #171952, #172345, and #172346).

#### Data and code availability

- The RNA-seq and Ribo-seq data have been deposited at GEO and are publicly available as of the date of publication. Accession numbers are listed in the key resources table.
- All original code has been deposited at GitHub and is publicly available as of the date of publication. DOIs are listed in the key resources table.
- Any additional information required to reanalyze the data reported in this paper is available from the lead contact upon request.

### EXPERIMENTAL MODEL AND STUDY PARTICIPANT DETAILS

#### Cell lines and culture conditions

Human 293T cells (female) were obtained from ATCC (CRL-3216; RRID:CVCL_0063). MB135 (female), MB135-iDUX4, MB135-iDUX4/ZSCAN4-mCherry, and MB200 (male) immortalized human myoblasts were a gift from Dr. Stephen Tapscott and originated from the Fields Center for FSHD and Neuromuscular Research at the University of Rochester Medical Center. MB135-iDUX4 cells have been described previously.^30^ MB135-iFLAG-SRSF3-FL, and MB135-iFLAG-SRSF3-TR immortalized human myoblasts were generated in this study. All parental cell lines were authenticated by karyotype analysis and determined to be free of mycoplasma by PCR screening. 293T cells were maintained in Dulbecco’s Modified Eagle Medium (DMEM) (Thermo Fisher Scientific) supplemented with 10% EqualFETAL (Atlas Biologicals). Myoblasts were maintained in Ham’s F-10 Nutrient Mix (Thermo Fisher Scientific) supplemented with 20% Fetal Bovine Serum (Thermo Fisher Scientific), 10 ng/mL recombinant human basic fibroblast growth factor (Promega), and 1 μM dexamethasone (Sigma-Aldrich). MB135-iDUX4/ZSCAN4-mCherry and MB135-iDUX4 myoblasts were additionally maintained in 2 μg/mL puromycin dihydrochloride (VWR). MB135-iFLAG-SRSF3-FL and -TR myoblasts were additionally maintained in 10 μg/mL blasticidin S HCl (Thermo Fisher Scientific). Induction of DUX4 and SRSF3 transgenes was achieved by culturing cells in 1–2 μg/mL doxycycline hyclate (Sigma-Aldrich). Differentiation of myoblasts into myotubes was achieved by switching the fully confluent myoblast monolayer into DMEM containing 1% horse serum (Thermo Fisher Scientific) and Insulin-Transferrin-Selenium (Thermo Fisher Scientific). All cells were incubated at 37°C with 5% CO_2_.

### METHOD DETAILS

#### Cloning

pTwist-FLAG-SRSF3_Full.Length_Codon.Optimized and pTwist-FLAG-SRSF3_Truncated_Codon.Optimized plasmids were synthesized by Twist Bioscience. To construct pCW57.1-FLAG-SRSF3_Full.Length_Codon.Optimized-Blast and pCW57.1-FLAG-SRSF3_Truncated_Codon.Optimized-Blast plasmids, the SRSF3 open reading frames were subcloned into pCW57-MCS1-P2A-MCS2 (Blast) (a gift from Adam Karpf, Addgene plasmid #80921)^53^ by restriction enzyme digest using EcoRI and BamHI (New England Biolabs).

#### Antibody generation

Purified SRSF3-TR peptide (Cys-PRRRVTIMSLLTTL) was used as an immunogen and polyclonal rabbit anti-SRSF3-TR antibody production was done in collaboration with Pacific Immunology (Ramona, CA). The antisera from all animals were screened for reactivity by ELISA against the immunogen and with western blots and immunofluorescence against transfected SRSF3-TR.

#### Transgenic cell line generation

Lentiviral particles expressing doxycycline-inducible FLAG-SRSF3-FL or -TR transgenes were generated by co-transfecting 293T cells with the appropriate lentivector, pMD2.G (a gift from Didier Trono, Addgene plasmid #12259), and psPAX2 (a gift from Didier Trono, Addgene plasmid #12260) using Lipofectamine 2000 Transfection Reagent (Thermo Fisher Scientific). To generate polyclonal SRSF3 transgenic cell lines, MB135 myoblasts were transduced with lentivirus in the presence of 8 μg/mL polybrene (Sigma-Aldrich) and selected using 10 μg/mL blasticidin S HCl.

#### Plasmid transfections

293T cells were transfected with pTwist-FLAG-SRSF3_Full.Length_Codon.Optimized and pTwist-FLAG-SRSF3_Truncated_Codon.Optimized plasmids using Lipofectamine 2000 Transfection Reagent following the manufacturer’s instructions.

#### Live cell imaging

MB135-iDUX4/ZSCAN4-mCherry myoblasts were induced with doxycycline hyclate to turn on DUX4 expression and subjected to time lapse imaging using the IncuCyte S3 incubator microscope system (Sartorius). Images were collected every 15 min from the time of doxycycline addition (t = 0 h) to 28 h.

#### RNA extraction and RT-qPCR

Total RNA was extracted from whole cells using TRIzol Reagent (Thermo Fisher Scientific) following the manufacturer’s instructions. Isolated RNA was treated with DNase I (Thermo Fisher Scientific) and reverse transcribed to cDNA using SuperScript III reverse transcriptase (Thermo Fisher Scientific) and random hexamers (Thermo Fisher Scientific) according to the manufacturer’s protocol. Quantitative PCR was carried out on a CFX384 Touch Real-Time PCR Detection System (Bio-Rad) using primers specific to each gene of interest and iTaq Universal SYBR Green Supermix (Bio-Rad). The expression levels of target genes were normalized to that of the reference gene *RPL27* using the delta-delta-Ct method.^54^ The primers used in this study are listed in the key resources table.

#### RNA-seq library preparation and sequencing

Total RNA was extracted from whole cells using TRIzol Reagent following the manufacturer’s instructions. Isolated RNA was subjected to ribosomal RNA depletion using the Ribo-Zero rRNA Removal Kit (Illumina). RNA-seq libraries were prepared using the NEXTflex Rapid Directional qRNA-Seq Kit (Bioo Scientific) following the manufacturer’s instructions and sequenced using 75 bp single-end sequencing on the Illumina NextSeq 500 platform by the BioFrontiers Institute Next-Gen Sequencing Core Facility.

#### Ribosome footprinting

Ribo-seq was performed as described previously^36^ using six 70% confluent 10 cm dishes of MB135-iDUX4 cells per condition. Briefly, cells were washed with ice-cold phosphate-buffered saline (PBS) supplemented with 100 μg/mL cycloheximide (Sigma-Aldrich), flash frozen on liquid nitrogen, and lysed in Lysis Buffer (PBS containing 1% (v/v) Triton X-100 and 25 U/mL TurboDNase (Ambion)). Cells were harvested by scraping and further lysed by trituration ten times through a 26-gauge needle. The lysate was clarified by centrifugation at 20,000 g for 10 min at 4°C. The supernatants were flash frozen in liquid nitrogen and stored at −80°C. Thawed lysates were treated with RNase I (Ambion) at 2.5 U/mL for 45 min at room temperature with gentle mixing. Further RNase activity was stopped by addition of SUPERaseIn RNase Inhibitor (Thermo Fisher Scientific). Next, ribosome complexes were enriched using MicroSpin S-400 HR Columns (GE Healthcare) and RNA extracted using the Direct-zol RNA Miniprep Kit (Zymo Research). Ribo-Zero rRNA Removal Kit was used to deplete rRNAs and the ribosome-protected fragments were recovered by running them in a 17% Urea gel, staining with SYBR Gold (Invitrogen), and extracting nucleic acids that are 27–30 nucleotides long from gel slices by constant agitation in 0.3 M NaCl at 4°C overnight. The recovered nucleic acids were precipitated with isopropanol using GlycoBlue Coprecipitant (Ambion) as carrier and treated with T4 polynucleotide kinase (Thermo Fisher Scientific). Libraries were prepared using the NEXTflex Small RNA-Seq Kit v3 (Bioo Scientific) following the manufacturer’s instructions and sequenced using 75 bp single-end reads on an Illumina NextSeq 500 by the BioFrontiers Institute Next-Gen Sequencing Core Facility.

#### RNA-seq and ribo-seq data analysis

Fastq files were stripped of the adapter sequences using cutadapt. UMI sequences were removed, and reads were collapsed to fasta format. Reads were first aligned against rRNA (accession number U13369.1), and to a collection of snoRNAs, tRNAs, and miRNA (retrieved using the UCSC table browser) using bowtie2.^55^ Remaining reads were mapped to the hg38 version of the genome (without scaffolds) using STAR 2.6.0a^56^ supplied with the GENCODE 25.gtf file. A maximum of two mismatches and mapping to a minimum of 50 positions was allowed. De novo splice junction discovery was disabled for all datasets. Only the best alignment per each read was retained. Quality control and read counting of the Ribo-seq data was performed with Ribo-seQC.^57^

Differential gene expression analysis of the RNA-seq data was conducted using DESeq2.^58^ Briefly, featureCounts from the subread R package^59^ was used to assign aligned reads (in BAM format) to genomic features supplied with the GENCODE 25. gtf file. The featureCounts output was then supplied to DESeq2 and differential expression analysis was conducted with the 0 h time point serving as the reference sample. Genes with very low read count were filtered out by requiring at least a total of 10 reads across the 12 samples (3 replicates each of the 0, 4, 8, and 14 h samples). Log2 fold change shrinkage was done using the apeglm function.^60^

Differential analysis of the RNA-seq and Ribo-seq data was performed using DESeq2, as previously described,^61,62^ using an interaction model between the tested condition and RNA-seq – Ribo-seq counts. Only reads mapping uniquely to coding sequence regions were used. In addition, ORFquant^36^ was used to derive de novo isoform-specific translation events, by pooling the Ribo-seQC output from all Ribo-seq samples, using uniquely mapping reads. DEXSeq^37^ was used to perform differential exon usage along the DUX4 time course data, using Ribo-seq counts on exonic bins and junctions belonging to different ORFquant-derived translated regions. NMD candidates were defined by ORFquant as open reading frames ending with a stop codon upstream of an exon-exon junction.

Profiles in Figure 3A were calculated using pairs of stop codons from transcripts of the same gene (PTC vs. canonical stop codon). A window of 100nt around stop codons was used, and only using genes with PTC windows not overlapping other coding regions. RNA-seq and Ribo-seq values over such windows were normalized: for each time point and assay (RNA-seq or Ribo-seq), library-depth-normalized values were divided by the total signal per gene (to account for different expression levels), and then 0–1 normalized; subsequently, the average of such profiles across genes were calculated.

#### GO category analysis

Gene Ontology (GO) analysis was conducted using the web tool http://geneontology.org, powered by pantherdb.org. Briefly, statistical overrepresentation test using the complete GO biological process annotation dataset was conducted and p values were calculated using the Fisher’s exact test and False Discovery Rate was calculated by the Benjamini-Hochberg procedure.

#### Polysome profiling

Polysome profiling was performed as previously described^63,64^ with the following modifications. Four 70% confluent 15 cm dishes of MB135-iDUX4 cells per condition were treated with 100 μg/mL cycloheximide for 10 min, transferred to wet ice, washed with ice-cold PBS containing 100 μg/mL cycloheximide, and then lysed in 400 μL Lysis Buffer (20 mM HEPES pH 7.4, 15 mM MgCl_2_, 200 mM NaCl, 1% Triton X-100, 100 μg/mL cycloheximide, 2 mM DTT, and 100 U/mL SUPERaseIn RNase Inhibitor) per 15 cm dish. The cells and buffer were scraped off the dish and centrifuged at 13,000 rpm for 10 min at 4°C. Lysates were fractionated on a 10%–60% sucrose gradient using the SW 41 Ti Swinging-Bucket Rotor (Beckman Coulter) at 36,000 rpm for 3 h and 10 min. Twenty-four fractions were collected using a Gradient Station ip (BioComp) and an FC 203B Fraction Collector (Gilson) with continuous monitoring of absorbance at 254 nm. RNA from each fraction was extracted using TRIzol LS Reagent (Thermo Fisher Scientific) following the manufacturer’s instructions. RT-qPCR was carried out as described above.

#### Protein extraction

Total protein was extracted from whole cells using TRIzol Reagent following the manufacturer’s instructions, excepting that protein pellets were dissolved in Protein Resuspension Buffer (0.5 M Tris base, 5% SDS). Isolated protein was quantified using the Pierce BCA Protein Assay Kit (Thermo Fisher Scientific) according to the manufacturer’s protocol. Protein was mixed with 4X NuPAGE LDS Sample Buffer (Thermo Fisher Scientific) containing 50 mM DTT and heated to 70°C before immunoblotting.

#### Immunoprecipitation

MB135-iDUX4 myoblasts were treated with or without doxycycline for 14 h and then trypsinized prior to lysis on ice in 1 mL of Lysis Buffer (50 mM Tris-HCl pH 7.5, 150 mM NaCl, 1% NP-40) containing protease inhibitors (Sigma Aldrich). Lysates were precleared using Protein G Sepharose (Thermo Fisher Scientific) for 1 h prior to an overnight incubation at 4°C with either anti-SRSF3 or anti-SRSF3-TR antibody. Protein G Sepharose was added the following morning for 5 h to bind the antibody, and beads were subsequently washed 5 times with 1 mL cold Lysis Buffer. After the final wash, 4X NuPAGE LDS Sample Buffer containing 50 mM DTT was added directly to the beads and samples heated to 70°C for protein elution before immunoblotting.

#### Immunoblotting

Protein was run on NuPAGE Bis-Tris precast polyacrylamide gels (Thermo Fisher Scientific) alongside PageRuler Plus Prestained Protein Ladder (Thermo Fisher Scientific) and transferred to Odyssey nitrocellulose membrane (LI-COR Biosciences). Membranes were blocked in Intercept (PBS) Blocking Buffer (LI-COR Biosciences) before overnight incubation at 4°C with primary antibodies diluted in Blocking Buffer containing 0.2% Tween 20. Membranes were incubated with IRDye-conjugated secondary antibodies (LI-COR Biosciences) for 1 h and fluorescent signal visualized using a Sapphire Biomolecular Imager (Azure Biosystems) and Sapphire Capture software (Azure Biosystems). When appropriate, membranes were stripped with Restore Western Blot Stripping Buffer (Thermo Fisher Scientific) before being re-probed. Band intensities were quantified by densitometry using ImageJ.^65^

#### Immunofluorescence

Cells were fixed in 10% Neutral Buffered Formalin (Research Products International) for 30 min and permeabilized for 10 min in PBS with 0.1% Triton X-100. Samples were then incubated overnight at 4°C with primary antibodies, followed by incubation with 488- or 594-conjugated secondary antibodies for 1 h prior to counterstaining and mounting with Prolong Diamond Antifade Mountant with DAPI (Thermo Fisher Scientific). Slides were imaged with a DeltaVision Elite deconvolution microscope, CoolSNAP HQ^2^ high-resolution CCD camera, and Resolve3D softWoRx-Acquire v7.0 software. ImageJ software^65^ was used for image analysis.

#### Solid phase synthesis of TMO chimeras (ASOs)

TMO chimeras were synthesized according to the previously reported procedure.^43,44^ Briefly, the 5′-dimethoxytrityl (DMT) protecting group of the solid supported 2′-deoxyribonucleoside (CPG-500 support, Glen Research) was deprotected in the first stage by using 3% trichloroacetic acid in dichloromethane. In the second stage, condensation of the resulting CPG-500 support linked 5′-hydroxyl-2′-deoxyribonucleoside with the 6′-DMT-morpholinonucleoside 3′-phosphordiamidites of mA^Bz^, mG^iBu^, mC^Bz^, mT (ChemGenes) or commercial 2′-deoxyribonucleoside 3′-phosphoramidites was achieved using 5-ethylthio-1H-tetrazole (ETT) in anhydrous acetonitrile as activator (30 s condensation time). Subsequent conversion of P(III) linkages to P(V) thiophosphoramidate (TMO) or P(V) 2′-deoxyribonucleoside 3′-thiophosphate was achieved by using 3-[(Dimethylaminomethylene)amino]-3H-1,2,4-dithiazole-5-thione (DDTT) as the sulfurization agent. Finally, the unreacted hydroxyl groups were acetylated by conventional capping reagents (Cap A: Tetrahydrofuran/Acetic Anhydride and Cap B: 16% 1-Methylimidazole in Tetrahydrofuran; Glen Research). The 5′-DMT protecting group on the resulting dinucleotide was next deprotected using deblocking mixture and this DMT deprotected dinucleotide was then used for additional cycles in order to generate ASOs having internucleotide thiophosphoramidate or thiophosphate linkages. The above cycle was repeated to provide the thiomorpholino oligonucleotide chimeras of the desired length and sequence. Cleavage of these 5′-protected DMT-on oligonucleotides from the solid support and deprotection of base and phosphorus protecting groups was carried out using 0.5 mL of 28% aqueous ammonia at 55°C for 16 h. Subsequently, the CPG was filtered through a micro spin centrifuge filter with pore size of 0.2 μm and the resulting filtrate was evaporated to dryness on a SpeedVac (Thermo Fisher Scientific). The residue was dissolved in 0.75 mL of 3% acetonitrile/water mixture and filtered through a micro spin centrifuge filter. A small portion of the crude sample was withdrawn and submitted to LCMS analysis. The remaining reaction mixture was purified by RP-HPLC. Fractions containing the pure ASO were combined, evaporated to dryness, and submitted to LCMS analysis. Fractions containing the pure DMT-on ASO were dissolved in 0.5 mL of detritylation mixture. After 25 min at 40°C, the mixture was neutralized with 5 μL of triethylamine, filtered using a micro spin centrifuge filter, and the filtrate containing the sample was purified by RP-HPLC column chromatography. Fractions containing the final DMT-off product were combined and evaporated to dryness on a SpeedVac. The residue was submitted to LCMS analysis in order to determine the purity of the sample. The concentration of the ASO was determined by NanoDrop spectrophotometry before storing the samples at −20°C.

#### LCMS analysis

LCMS analysis was performed on an Agilent 6530 series Q-TOF LC/MS spectrometer. A Waters ACQUITY UPLC BEH C18, 1.7 μm, 2.1 × 100 nm column was used as the stationary phase. Aqueous phase was Buffer A (950 mL water, 25 mL methanol, 26 mL hexafluoro-2-propanol (HFIP) and 2.5 mL triethyl amine) and organic phase was Buffer B (925 mL methanol, 50 mL water, 26 mL hexafluoro-2-propanol (HFIP) and 2.5 mL triethyl amine). The gradient was 0–100% of Buffer B for 30 min followed by 100% Buffer B for 5 min at a flow rate of 0.2 mL/min and a set temperature of 25°C. The observed masses of the ASOs were consistent with the expected theoretical masses.

#### Antisense oligonucleotide transfections

ASOs were transfected into MB135-iDUX4 cells 40 h prior to doxycycline induction using Lipofectamine RNAiMAX Transfection Reagent (Thermo Fisher Scientific) following the manufacturer’s instructions. The ASOs used in this study are listed in the key resources table.

#### Cell viability assays

Caspase 3/7 activity was used to determine cell viability. MB135-iFLAG-SRSF3-FL and -TR cells were seeded in 96-well plates at 2.5e3 cells per well and treated with 1 μg/mL doxycycline hyclate to induce transgene expression. MB135-iDUX4 cells were seeded in 24-well plates at 8e4 cells per well, transfected with ASOs as described above, and 40 h later treated with 2 μg/mL doxycycline hyclate or seeded in 96-well plates at 3e3 cells per well and 24 h later treated with 1 μg/mL doxycycline hyclate and either DMSO or 10 μM MG132 (Sigma-Aldrich). Caspase 3/7 activity was measured 16 and 24 h later using the Caspase-Glo 3/7 Assay System (Promega) following the manufacturer’s instructions. Luminescence was detected using a GloMax-Multi Detection System (Promega).

#### Antibodies

The antibodies used in this study are anti-DUX4 (Abcam 124699), anti-eIF2α (Santa Cruz Biotechnology sc-133132), anti-phosphoe-IF2α (Abcam ab32157), anti-Histone H3 (Abcam 1791), anti-SRSF3 (Thermo Fisher Scientific 33–4200), anti-SRSF3-TR (this paper), anti-RENT1/hUPF1 (Abcam ab109363), Drop-n-Stain CF 488A Donkey Anti-Rabbit IgG (Biotium 20950), Drop-n-Stain CF 594 Donkey Anti-Rabbit IgG (Biotium 20951), IRDye 650 Goat anti-Mouse IgG Secondary Antibody (LI-COR Biosciences 926–65010), and IRDye 800CW Goat anti-Rabbit IgG Secondary Antibody (LI-COR Biosciences 926–32211).

### QUANTIFICATION AND STATISTICAL ANALYSIS

#### Data analysis, statistical tests, and visualization

All data analysis and statistical tests were performed in the R programming environment and relied on Bioconductor^66^ and ggplot2.^67^ Statistical details of specific experiments can be found in the results, STAR Methods, and/or Figure Legends. Plots were generated using R plotting functions and/or the ggplot2 package. Bar graphs were generated using GraphPad Prism software version 9.0. Biological replicates were defined as experiments performed separately on distinct samples (i.e. cells cultured in different wells) representing identical conditions and/or time points. No outliers were eliminated in this study.

## Supplementary Material

1

2

3

4

5

## Figures and Tables

**KEY RESOURCES TABLE:** 

REAGENT or RESOURCE	SOURCE	IDENTIFIER

Antibodies		

Rabbit monoclonal anti-DUX4 (clone E5-5)	Abcam	Cat#ab124699; RRID:AB_10973363
Mouse monoclonal anti-eIF2α (clone D-3)	Santa Cruz Biotechnology	Cat#sc-133132; RRID:AB_1562699
Rabbit monoclonal anti-phospho-eIF2α (clone E90)	Abcam	Cat#ab32157; RRID:AB_732117
Rabbit polyclonal anti-Histone H3	Abcam	Cat#ab1791; RRID:AB_302613
Mouse monoclonal anti-SRSF3 (clone 7B4 (7B4A12))	Thermo Fisher Scientific	Cat#33–4200; RRID:AB_2533119
Rabbit polyclonal anti-SRSF3-TR	This paper	N/A
Rabbit monoclonal anti-RENT1/hUPF1 (clone EPR4681)	Abcam	Cat#ab109363; RRID:AB_10861979

Chemicals, peptides, and recombinant proteins		

Insulin-Transferrin-Selenium	Thermo Fisher Scientific	Cat#41400045
Protein G Sepharose	Thermo Fisher Scientific	Cat#101241
Recombinant human basic fibroblast growth factor	Promega	Cat#G5071, discontinued
Blasticidin S HCl	Thermo Fisher Scientific	Cat#R21001
Cycloheximide	Sigma-Aldrich	Cat#239765
Doxycycline hyclate	Sigma-Aldrich	Cat#D9891
Lipofectamine 2000 Transfection Reagent	Thermo Fisher Scientific	Cat#11668-030
Lipofectamine RNAiMAX Transfection Reagent	Thermo Fisher Scientific	Cat#13778-150
MG132	Sigma-Aldrich	Cat#474790
Puromycin dihydrochloride	VWR	Cat#97064-280
TRIzol Reagent	Thermo Fisher Scientific	Cat#15596018

Critical commercial assays		

BCA Protein Assay Kit	Pierce	Cat#23225
Caspase-Glo 3/7 Assay System	Promega	Cat#G8091
Direct-zol RNA Miniprep Kit	Zymo Research	Cat#R2051
NEXTflex Rapid Directional qRNA-Seq Kit	Bioo Scientific	Cat# NOVA-5130-02D
NEXTflex Small RNA-Seq Kit v3	Bioo Scientific	Cat# NOVA-5132-06
Ribo-Zero rRNA Removal Kit	Illumina	Cat# MRZH11124, discontinued
SuperScript III First-Strand Synthesis System	Thermo Fisher Scientific	Cat#18080051

Deposited data		

RNA-seq and Ribo-seq data	This paper	GEO: GSE178761

Experimental models: Cell lines		

Human: 293T	ATCC	CRL-3216; RRID:CVCL_0063
Human: MB135 myoblasts	Laboratory of Stephen Tapscott	N/A
Human: MB135-iDUX4 myoblasts	Laboratory of Stephen Tapscott^30^	N/A
Human: MB135-iDUX4/ZSCAN4-mCherry myoblasts	Laboratory of Stephen Tapscott	N/A
Human: MB135-iFLAG-SRSF3-FL myoblasts	This paper	N/A
Human: MB135-iFLAG-SRSF3-TR myoblasts	This paper	N/A
Human: MB200 myoblasts	Laboratory of Stephen Tapscott	N/A

Oligonucleotides		

Primers for qPCR, see Table S4	This paper	N/A
Antisense oligonucleotide: SRSF3_TMO-11: G*A*T*G*G*t*g*a*c*t*c*t*g*c*g*A*C*G*A*g (*, thiophosphoramidate or thiophosphate internucleotide linkage; capital letter, morpholino nucleoside; lowercase letter, 2’deoxynucleoside)	This paper	N/A

Recombinant DNA		

Plasmid: pCW57.1-FLAG-SRSF3_Full.Length_Codon.Optimized-Blast	This paper	Addgene Plasmid #171951
Plasmid: pCW57.1-FLAG-SRSF3_Truncated_Codon.Optimized-Blast	This paper	Addgene Plasmid #171952
Plasmid: pCW57-MCS1-P2A-MCS2 (Blast)	Laboratory of Adam Karpf^53^	Addgene Plasmid #80921; RRID:Addgene_80921
Plasmid: pMD2.G	Laboratory of Didier Trono	Addgene Plasmid #12259; RRID:Addgene_12259
Plasmid: psPAX2	Laboratory of Didier Trono	Addgene Plasmid #12260; RRID:Addgene_12260s
Plasmid: pTwist-FLAG-SRSF3_Full.Length_Codon.Optimized	This paper	Addgene Plasmid #172345
Plasmid: pTwist-FLAG-SRSF3_Truncated_Codon.Optimized	This paper	Addgene Plasmid #172346

Software and algorithms		

Code used for RNA-seq and Ribo-seq figure generation	This paper	https://github.com/sjaganna/2021-campbell_dyle_calviello_et_al; https://doi.org/10.5281/zenodo.7951674
GraphPad Prism	GraphPad Prism	https://graphpad.com; RRID:SCR_002798
ImageJ	ImageJ	https://imagej.nih.gov; RRID:SCR:_003070

## Highlights

- Paired RNA-seq and ribosome profiling following time course of DUX4 expression
- DUX4-expressing cells show concordance of transcript levels and translation status
- Loss of NMD by DUX4 causes widespread production of truncated RBPs
- The truncated splicing factor SRSF3 is cytotoxic
