# Supplementary material for: Compromised nonsense-mediated RNA decay results in truncated RNA-binding protein production upon DUX4 expression
Source: Cell Rep. Author manuscript; Available in PMC 2023 Oct 23. (PMC10592454; doi:10.1016/j.celrep.2023.112642)
Supplement: 1 [file NIHMS1912796-supplement-1.pdf]

Cell Reports, Volume *42*

Supplemental information

Compromised nonsense-mediated RNA decay results in truncated RNA-binding protein production upon DUX4 expression

Amy E. Campbell, Michael C. Dyle, Roberto Albanese, Tyler Matheny, Kavitha Sudheendran, Michael A. Cortázar, Thomas Forman, Rui Fu, Austin E. Gillen, Marvin H. Caruthers, Stephen N. Floor, Lorenzo Calviello, and Sujatha Jagannathan

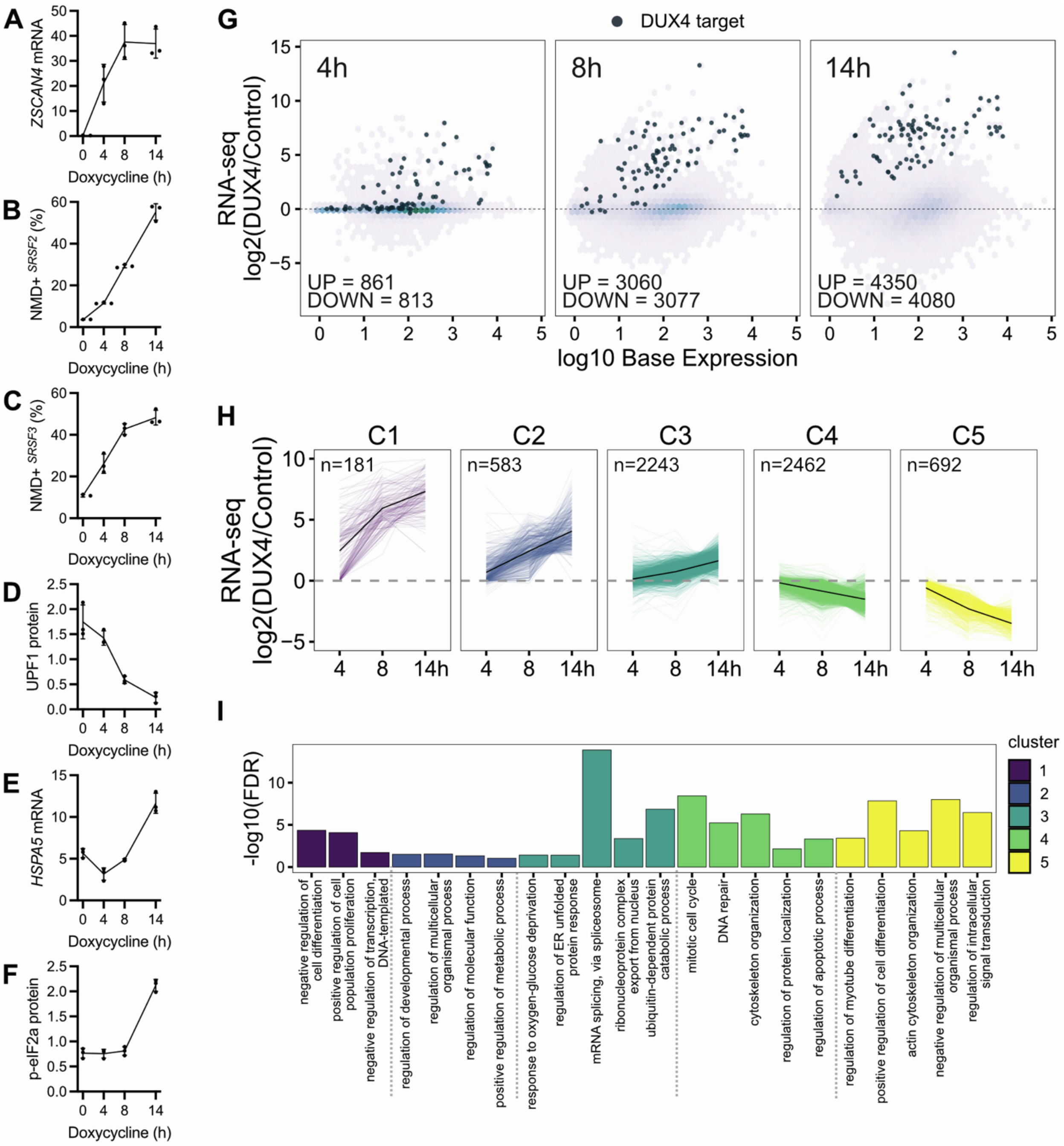

Figure S1. Time course RNA-seq in MB135-iDUX4 myoblasts reveals early transcript-level changes in pathways underlying FSHD pathology, Related to Figure 1. (**A**) Levels of DUX4 target gene *ZSCAN4* mRNA as normalized to *RPL27* expression determined by RT-qPCR. (**B-C**) Isoform ratios of NMD-targeted isoforms of *SRSF2* (**B**) and *SRSF3* (**C**) mRNA as determined by RT-qPCR. (**D**) Quantification of UPF1 protein levels from immunoblotting of total protein as normalized to GAPDH loading control. (**E**) RNA levels of *HSPA5*, a marker of the unfolded protein response, as normalized to *RPL27* expression determined by RT-qPCR. (**F**) Quantification of phosphorylated eIF2α protein levels from immunoblotting of total protein as normalized to total eIF2α protein levels. (**G**) M-A plots for RNA-seq data after 4, 8, and 14 h of DUX4 induction compared to the 0 h control. DUX4 target status defined as in[1]. (**H**) Log2 fold change in RNA

expression from the 0 h time point is shown for each gene after k-means clustering. The thick black line represents the cluster mean. (**I**) GO analysis results of selected gene sets (biological process complete) that are significantly enriched in each cluster defined in (**H**). All error bars denote the standard deviation from the mean of three biological replicates, which are shown as individual data points.

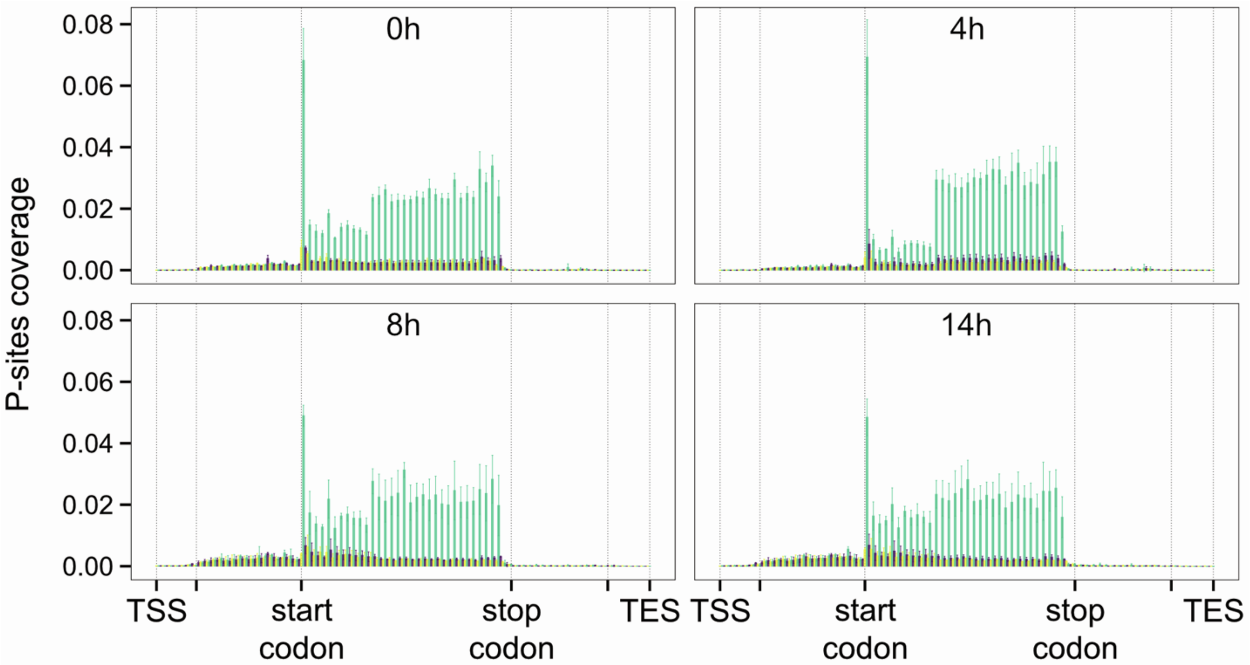

Figure S2. Ribo-seq quality control, Related to Figure 2. Aggregate profile of P-sites coverage (as calculated by Ribo-seQC[2]) depicting single nucleotide resolution of Ribo-seq data along the time course. Each frame is shown with a different color. Error bars represent the standard deviation from three biological replicates.

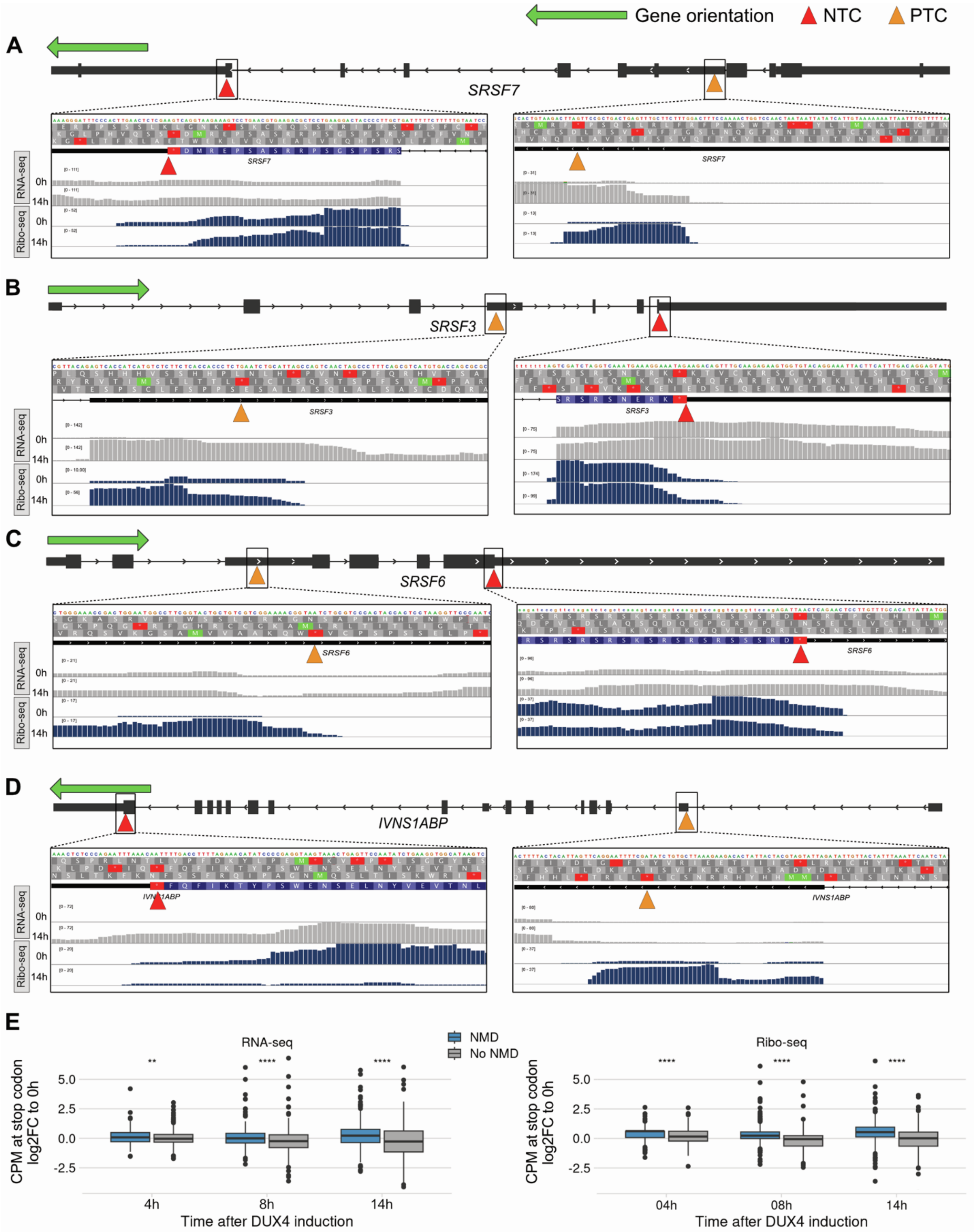

Figure S3. Increased abundance and translation of NMD-targeted transcripts, Related to Figure 3. (**A-D**) Example screenshots displaying RNA-seq and Ribo-seq tracks around PTCs detected by ORFquant[3]. (**E**) Count Per Million (CPM) log2 fold changes to 0 h of RNA-seq and

Ribo-seq signals around stop codons are shown on the y-axis across a time course of DUX4 induction. One-sided paired Wilcoxon test was used to assess statistical significance. For the log2FC calculation, a pseudocount was added to each CPM.

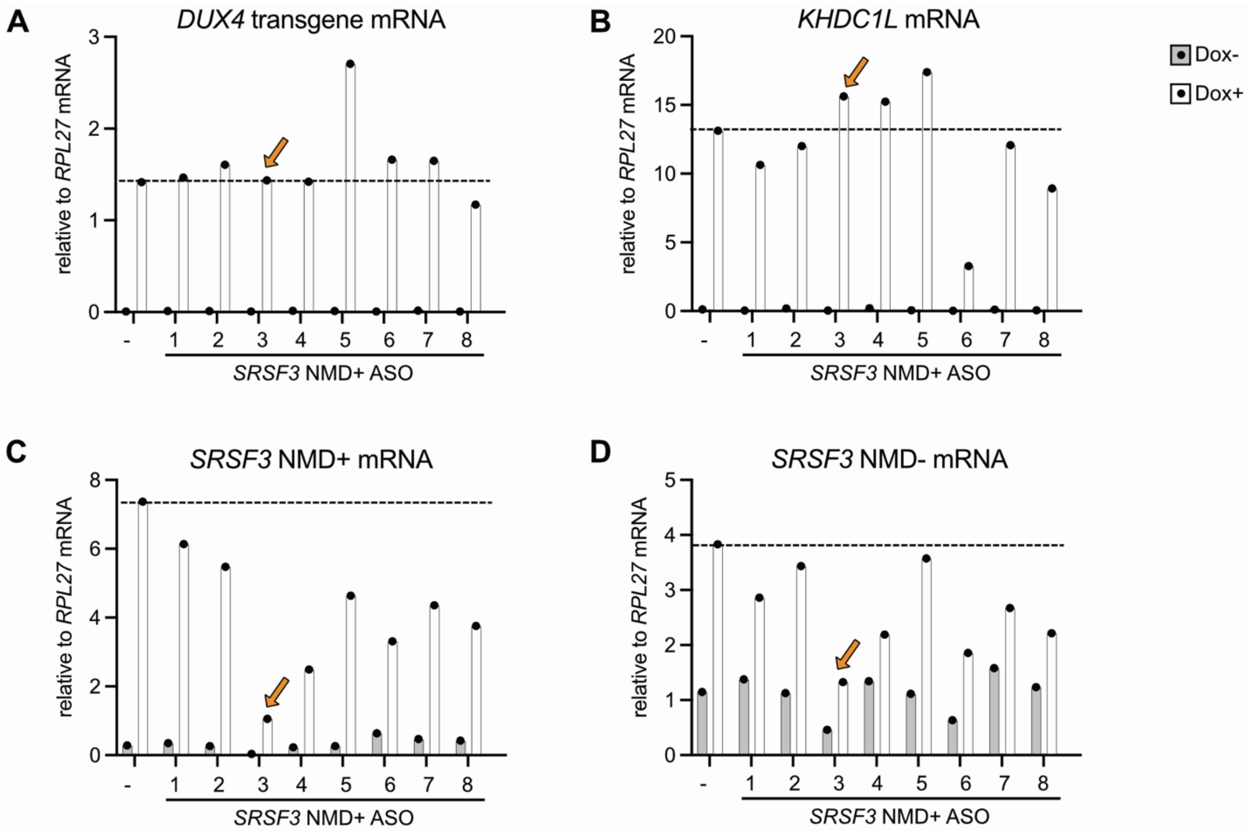

Figure S4. qRT-PCR confirmation of ASO knockdowns, Related to Figure 4. Relative RNA levels of (**A**) *DUX4* transgene, (**B**) *KHDC1L* (DUX4 target gene), (**C**) *SRSF3* NMD+, and (**D**) *SRSF3* NMD- isoforms as determined by RT-qPCR following transfection with antisense oligonucleotides (ASOs) targeting *SRSF3* NMD+ and treatment without (-) or with (+) doxycycline (Dox) for 14 h to induce DUX4 in MB135-iDUX4 myoblasts. The tested ASOs are numbered 1-8; "-" indicates the no ASO control. The orange arrow indicates the ASO chosen for further studies in Figure 4F.

Table S4. List of qPCR primers used in this study, Related to STAR Methods.

Primer name	Primer sequence	Source
DUX4 transgene F	TAGGGGAAGAGGTAGACGGC	DOI: 10.1093/hmg/ddz242
DUX4 transgene R	CGGTTCCGGGATTCCGATAG	DOI: 10.1093/hmg/ddz242
HSPA5 F	CGGGCAAAGATGTCAGGAAAG	DOI: 10.1126/science.1146361
HSPA5 R	TTCTGGACGGGCTTCATAGTAGAC	DOI: 10.1126/science.1146361
KHDC1L F	CACCAATGGCAAAGCAGTGG	DOI: 10.1093/hmg/ddw271
KHDC1L R	TCAGTCTCCGGTGTACGGTG	DOI: 10.1093/hmg/ddw271
SRSF2 F	GTGTCCAAGAGGGAATCCAA	DOI: 10.7554/eLife.04996
SRSF2 NMD- R	TGCTTGCCGATACATCATTT	DOI: 10.7554/eLife.04996
SRSF2 NMD+ R	AGGAGACCGCAGCATTTTCT	DOI: 10.7554/eLife.04996
SRSF3 F	TGGAACTGTCGAATGGTGAA	DOI: 10.7554/eLife.04996
SRSF3 NMD- R	CTTGGAGATCTGCGACGAG	DOI: 10.7554/eLife.04996
SRSF3 NMD+ R	GGGTGGTGAGAAGAGACATGA	DOI: 10.7554/eLife.04996
RPL27 F	GCAAGAAGAAGATCGCCAAG	DOI: 10.1093/hmg/ddw271
RPL27 R	TCCAAGGGGATATCCACAGA	DOI: 10.1093/hmg/ddw271
ZSCAN4 F	TGGAAATCAAGTGGCAAAAA	DOI: 10.1016/j.devcel.2011.11.013
ZSCAN4 R	CTGCATGTGGACGTGGAC	DOI: 10.1016/j.devcel.2011.11.013

SUPPLEMENTAL REFERENCES

1. Yao, Z., Snider, L., Balog, J., Lemmers, R.J., Van Der Maarel, S.M., Tawil, R., and Tapscott, S.J. (2014). DUX4-induced gene expression is the major molecular signature in FSHD skeletal muscle. Hum Mol Genet *23*, 5342-5352. https://doi.org/10.1093/hmg/ddu251.

2. Harnett, D., Meerdink, E., Calviello, L., Sydow, D., and Ohler, U. (2021). Genome-wide analysis of actively translated open reading frames using RiboTaper/ORFquant. Methods Mol Biol *2252*, 331-346. doi: 10.1007/978-1-0716-1150-0_16.

3. Calviello, L., Hirsekorn, A., and Ohler, U. (2020). Quantification of translation uncovers the functions of the alternative transcriptome. Nat Struct Mol Biol *27*, 717-725. https://doi.org/10.1038/s41594-020-0450-4.
